# The Impact of Comment Slant and Comment Tone on Digital Health Communication Among Polarized Publics: A Web-Based Survey Experiment

**DOI:** 10.2196/57967

**Published:** 2024-11-15

**Authors:** Fangcao Lu, Caixie Tu

**Affiliations:** 1 Department of Applied Social Sciences, Faculty of Health and Social Sciences, The Hong Kong Polytechnic University Kowloon, Hong Kong China (Hong Kong); 2 School of Journalism and Communication, Shanghai University Shanghai China

**Keywords:** comments slant, incivility, social media, influence of presumed influence, health compliance, mask wearing, web survey

## Abstract

**Background:**

Public attitudes toward health issues are becoming increasingly polarized, as seen in social media comments, which vary from supportive to oppositional and frequently include uncivil language. The combined effects of comment slant and comment tone on health behavior among a polarized public need further examination.

**Objective:**

This study aims to examine how social media users’ prior attitudes toward mask wearing and their exposure to a mask-wearing–promoting post, synchronized with polarized and hostile discussions, affect their compliance with mask wearing.

**Methods:**

The study was a web-based survey experiment with participants recruited from Amazon Mechanical Turk. A total of 522 participants provided consent and completed the study. Participants were assigned to read a fictitious mask-wearing–promoting social media post with either civil anti–mask-wearing comments (130/522, 24.9%), civil pro–mask-wearing comments (129/522, 24.7%), uncivil anti–mask-wearing comments (131/522, 25.1%), or uncivil pro–mask-wearing comments (132/522, 25.3%). Following this, the participants were asked to complete self-assessed questionnaires. The PROCESS macro in SPSS (model 12; IBM Corp) was used to test the 3-way interaction effects between comment slant, comment tone, and prior attitudes on participants’ presumed influence from the post and their behavioral intention to comply with mask-wearing.

**Results:**

Anti–mask-wearing comments led social media users to presume less influence about others’ acceptance of masks (B=1.49; *P*<.001; 95% CI 0.98-2.00) and resulted in decreased mask-wearing intention (B=0.07; *P*=.03; 95% CI 0.01-0.13). Comment tone with incivility also reduced compliance with mask-wearing (B=–0.44; *P*=.02; 95% CI –0.81 to –0.07). Furthermore, polarized attitudes had a direct impact (B=0.86; *P*<.001; 95% CI 0.45-1.26) and also interacted with both the slant and tone of comments, influencing mask-wearing intention.

**Conclusions:**

Pro–mask-wearing comments enhanced presumed influence and compliance of mask-wearing, but incivility in the comments hindered this positive impact. Antimaskers showed increased compliance when they were unable to find civil support for their opinion in the social media environment. The findings suggest the need to correct and moderate uncivil language and misleading information in online comment sections while encouraging the posting of supportive and civil comments. In addition, information literacy programs are needed to prevent the public from being misled by polarized comments.

## Introduction

### Background

Escalating political polarization is increasingly reflected in public attitudes toward health issues, despite health traditionally being a nonpartisan, science-based sector [1]. This polarization is particularly noticeable in social media discussions, where health-related posts often elicit a spectrum of public responses in the comments section, ranging from support to opposition [2,3]. Alarmingly, these comments frequently include incivility [4], such as profanity, name-calling, or shouting [5]. Particularly, social media has become a crucial tool for health communication, allowing health institutions to initiate campaigns and individual users to disseminate these campaign messages [6]. The prevalence of polarization and incivility in the comments accompanying the ubiquitous health campaigns on social media necessitates an understanding of their impact on health-related compliance behavior. This understanding is crucial for guiding public health promotion and enhancing the effectiveness of digital health communication.

Previous research has indicated that individuals’ exposure to opposing or uncivil comments in health promotion posts can independently reduce their compliance with the promoted health behaviors [7-9]. However, few studies have examined how the 2 attributes of comments interact and exert joint effects. Indeed, incivility might reduce the effects of comment slant as individuals may attribute low credibility to the commenters [10], thereby being less affected by them. The combined influence of comment slant and tone on health-related compliance behavior warrants an examination, as these 2 attributes of comments often occur together [11,12]. The findings contribute to a nuanced understanding of how social media users’ interaction and active participation, specifically polarized and hostile online discourse, affect digital health practices.

Health campaigns typically influence compliance behavior indirectly, rather than directly. The influence of presumed influence (IPI) model provides a theoretical explanation for this indirect effect, suggesting that individuals’ perceived media influence on others drives their compliance with promoted behaviors [13]. Even on social media, where cues (eg, view counts and comments) directly trigger normative influence to affect individuals [14], the IPI process remains significant for understanding their behavior [15]. Studies primarily suggest that individuals’ perceptions of a message’s influence on others are based on their assumptions about others’ exposure to that message [16,17]. This other-consciousness perspective highlights the effects of comments as they represent others’ responses to a post. Therefore, it is essential to explore how user comments with varying slants and tones can cultivate individuals’ perceptions of the campaign message’s influence on others and their compliance with health behaviors.

In a polarized environment, individuals often have strong prior attitudes. These polarized attitudes can also influence how they perceive the media’s impact on others, an approach known as the self-centered perspective of the IPI process [15]. While studies have started to delve into the effects of the self-centered perspective on the IPI process, whether and how this perspective introduces changes in the current predominant other-consciousness perspective remains unexplored. Notably, individuals may react differently to incivility in comments, showing more tolerance for comments that align with their opinions [18,19]. It implies that an individual’s polarized attitude can influence not only their perceived influence of the digital health campaign on others but also the effects of comments on their presumed influence of the campaign. By considering the separate and combined effects of the other-consciousness and self-centered perspectives, the dual nature of the IPI model offers a comprehensive understanding of the social psychological process through which people respond to mediated health communication in a polarized and hostile online environment.

This study was conducted in September 2020, during the early stage of the COVID-19 pandemic in the United States, when wearing masks to combat COVID-19 was controversial due to the polarized political environment [20]. Although numerous posts on social media advocated mask-wearing as an effective measure against the virus’s spread, comments on these posts were predominantly polarized [21], and about 1 in 5 of these comments exhibited incivility [22]. Such opinion climates have been related to the public’s noncompliance with COVID-19 mitigation guidelines [23], which leads to heightened virus transmission and COVID-19–related deaths. This situation provides an appropriate context to examine the impact of digital health promotion in the polarized and hostile digital space. Given that, we conducted a between-subjects experiment with a 2 (comment slant: pro–mask-wearing vs anti–mask-wearing) × 2 (comment tone: civil vs uncivil) design by manipulating comments accompanying a social media post for mask-wearing. Participants’ prior attitude was included as a moderator. Given the proliferation of digital health campaigns and the increasing polarized and hostile opinion climates, public health practitioners can benefit from the findings to boost the effectiveness of digital health communication.

### Presumed Influence and Health Campaigns

The IPI model comprises 3 components: presumed exposure, presumed influence, and the IPI [13]. *Presumed exposure* refers to individuals’ exposure to media content serving as a foundation for their inference about others’ exposure to the same content. *Presumed influence* indicates that, in turn, individuals’ presumption of others’ exposure triggers their presumption that the media content will influence those others. Finally, *IPI* refers to individuals’ alignment of their reactions to the presumed influence on others.

To date, discussions on IPI tend to focus on how people accommodate or rectify media messages. Rectifying behavior refers to individuals taking actions to protect others from harmful media effects or to magnify desirable media effects on others [24]. Accommodation reactions are more widely studied in the context of health campaigns, where individuals adapt themselves to the social environment [24]. To assess group or social norms, people often form perceptions about media influence on others and draw conclusions based on these perceived influences [25,26]. The more individuals believe that others adopt a particular behavior, the more likely they are to think that the behavior is normative. A desire to fit in with the group or social pressure then motivates them to adopt the same behavior [27,28]. The IPI model has been extensively tested in the context of health communication. The compliance behavior has been examined in the context of condom use, healthy diet, regular exercise, antismoking, excessive drinking, e-cigarette use, and COVID-19 pandemic protective behavior [25-28].

### Other-Consciousness Perspective: Comments and Presumed Influence

#### Overview

Social media has served as an integral arena for organizations and individuals to share health information [6]. The commentary feature provided by social media transforms audiences from passive information receivers to active users who interact with these health messages. Comments on a public health campaign message often reflect commenters’ support or opposition to the message, indicating their slants. Comments can express approval of the campaign by presenting supportive views or can be disapproving by presenting challenging views [29,30]. The slants of comments accompanying a message are likely to affect people’s presumptions of the message’s influence on others [7]. This effect can be explained by the exemplification theory. According to the theory, exemplars refer to the opinions or experiences of a person involved in an issue [31]. Exemplars are concrete and easy to process and remember. Thus, people tend to form judgments and beliefs about an issue based on available exemplars. Comments below a message serve as vivid exemplars of the audience’s opinions on the message. When gauging the influence of a social media message on others, a person may perceive comments below the message as representations of the entire audience’s reaction to the message [32,33].

Previous studies have found that the slant of comments accompanying social media health campaigns affects individuals’ perceptions of the campaigns’ influence on others. When individuals were exposed to supportive comments below a Facebook post promoting COVID-19 vaccination, they perceived a greater influence of the post on others’ acceptance of COVID-19 vaccination than when exposed to disapproving comments about the post [7]. Similarly, when social media users encounter pro–mask-wearing comments rather than anti–mask-wearing comments below a mask-promoting post, they are likely to perceive more influence of the post on other users’ acceptance of mask-wearing. The perceived influence of such a media message on others may further lead the users to comply with the behavior promoted by the message [34]. Accordingly, the following hypotheses are proposed:

- Hypothesis 1a: social media users will have weaker intentions to wear masks when exposed to anti–mask-wearing comments below a mask-promoting post than when exposed to pro–mask-wearing comments.
- Hypothesis 1b: the association between comment slants and intentions to wear masks will be mediated by social media users’ perception of the influence of the mask-promoting post on others.

Although the commentary feature facilitates social media users’ expressions of personal opinions, comments are often loaded with incivility. Comments are considered to contain incivility when expressed in an impolite and disrespectful tone [5]. Uncivil comments associated with a message can induce a “nasty effect,” a belief that if comments below a message contain incivility, the message must be bad [35]. Readers tend to believe that the original post, juxtaposed with the uncivil comments, is biased, of low quality, uncivil, and from a noncredible source [35-38].

Research on the “nasty effect” has also extended the spillover effects of comments’ incivility to audiences’ perception of a media message’s influence on others. Waddell and Bailey [39] found a belief in audiences’ minds that “if others’ comments are uncivil then they must not have been affected by the content.” Uncivil comments reveal conflicts among people with different opinions on an issue, rather than their elaboration and information processing of the issue discussed in the main message. When exposed to uncivil comments rather than civil ones left on a media message, people tend to believe that others reinforce their prior views rather than reading, deliberating, and being influenced by the adjacent media message. Accordingly, social media users exposed to uncivil comments on a mask-promoting post are expected to presume that the post exerts less influence on others’ acceptance of mask-wearing than when exposed to civil comments. The perception of less influence of the post on others, in turn, reduces social media users’ behavioral intention to wear masks. We thus propose the following hypotheses.

- Hypothesis 2a: social media users will have weaker intentions to wear masks when exposed to uncivil comments below a mask-promoting post than when exposed to civil comments.
- Hypothesis 2b: the association between comment tone and intentions to wear masks will be mediated by social media users’ perception of the influence of the mask-promoting post on others.

#### Self-Centered Perspective: Polarized Attitudes and Presumed Influence

Individuals’ perceptions of a health campaign’s influence on others may be affected by their prior attitudes toward the campaign’s advocacy. This effect can be explained by the “looking-glass perception,” which suggests that people’s social perceptions are often self-centric, and people tend to use their own opinions to estimate those of others [40,41]. They believe that situational factors are similar between themselves and others. Therefore, they tend to amplify their prior attitudes to their perceived social consensus on related issues [42].

Previous studies have provided support for the idea that presumed influence may be self-centric. For example, the robust causal chain from self-exposure, presumed exposure, to presumed influence was found to result from the order of questions. When the order of questions (self-variable → other variable → presumed influence on others → behavior) was reversed (other variable → self-variable → presumed influence on the self → behavior), the causal chain conflicted with the IPI process [43]. The finding suggests that the self may serve as an anchor for projecting presumed influence on others. Another study found that the more individuals relate themselves to the message and consider it real, the greater they perceive the message to elicit an influence on its audiences [15].

Extrapolating from this self-centered perspective, individuals’ prior attitudes toward a health campaign’s advocacy may predict their estimation of the campaign’s influence on others. People tend to accept information that is consistent with their prior beliefs [44]. When individuals encounter a health message consistent with their prior attitudes, they are more willing to acknowledge that they are influenced by the message and accept its view. In contrast, individuals are more likely to reject the message when they have inconsistent attitudes toward it [45-47]. Accordingly, individuals with favorable attitudes toward mask-wearing are likely to perceive that others, like themselves, also agree with the mask-promoting message and will be influenced by it. Individuals with unfavorable attitudes toward mask-wearing are likely to believe that others, similar to themselves, reject the message and are immune to it. The perception that the mask-promoting post has affected others, in turn, influences individuals’ behavioral intention to wear masks.

In addition to the partial mediating role of presumed influence, the positive association between attitudes and behavioral intentions has been sufficiently addressed [48]. Attitudes toward a health behavior can inspire individuals’ intention to perform the behavior. Thus, the following 2 hypotheses are proposed:

- Hypothesis 3a: social media users will have weaker intentions to wear masks when they have unfavorable attitudes toward mask-wearing than when they have favorable attitudes.
- Hypothesis 3b: the association between prior attitudes toward mask-wearing and intentions to wear masks will be partially mediated by social media users’ perception of the influence of the mask-promoting post on others.

#### The Interaction of Social Media Comments and Polarized Attitudes

The slant and tone of social media comments below a mask-promoting message are likely to interact and affect social media users’ presumption of the message’s influence and subsequently their health compliance. The content of the message provides important cues that help users form impressions of the senders. Previous studies reveal that encountering uncivil comments under a news article led to negative perceptions and less perceived credibility of the commenters [10]. While source credibility has long been recognized as a key factor in persuasiveness [49], a lack of credibility among commenters may cause uncivil comments to signal that the message has less influence on others. The reduced presumed influence, in turn, is less likely to drive behavioral change.

In other words, social media users’ exposure to civil pro–mask-wearing comments on a mask-wearing post facilitates their perception that the post poses an influence on others’ acceptance of mask-wearing and stimulates their compliance with mask-wearing. In contrast, exposure to uncivil pro–mask-wearing comments on a post is likely to decrease this presumed influence of the post and their intentions to comply with mask-wearing. In addition, social media users’ exposure to civil anti–mask-wearing comments on a mask-wearing post can reduce their presumed influence of the post on others’ acceptance of mask-wearing and their compliance with mask-wearing. Conversely, uncivil anti–mask-wearing comments can offset these negative effects to some extent by maintaining the social media users’ presumed influence and behavioral intention of mask-wearing. The following 2 hypotheses are therefore proposed:

- Hypothesis 4a: comment tone will moderate the effect of comment slant on social media users’ intentions to wear masks, such that the effect of comment slant on behavioral intention will be stronger when comments are expressed in a civil manner compared to in an uncivil manner.
- Hypothesis 4b: the interaction effect of comment slant and comment tone on social media users’ intentions to wear masks will be mediated by their perception of the influence of the mask-promoting post on others.

It is also likely that there is an interaction between comment slant, comment tone, and prior attitudes. Individuals may have more tolerance for comments that align with their prior attitudes, and they may overlook the incivility and aggressiveness in the comments [19]. This can be explained by the Social Identity Theory [50], which posits that individuals categorize themselves and others into in groups and out groups based on shared characteristics or beliefs, and this categorization influences their attitudes and behaviors. Individuals may categorize comments into in-group comments (those that align with their prior attitudes) and out-group comments (those that contradict their prior attitudes). They are more likely to favor in-group comments and perceive them in a positive light (ie, less uncivil) than out-group comments, as these comments reinforce their social identity and validate their prior attitudes.

Experimental studies have suggested that individuals would rate a comment that supported their prior attitudes as civil, even though it contained incivility. However, they still recognized the incivility in comments that were against their prior attitudes [18]. Thus, comment tone may only function or produce a relatively greater effect on presumed influence and health-related compliance behavior when social media users’ prior attitudes are inconsistent with comment slant. In contrast, when social media users’ prior attitudes are consistent with comment slant, they may ignore the incivility in these comments but perceive it as civil. Therefore, the impact of comment tone on presumed influence and behavioral intention would be discounted or become nonsignificant. We propose the 2 hypotheses below:

- Hypothesis 5a: there is an interaction among comment slant, comment tone, and prior attitudes on social media users’ intentions to wear masks, such that the influence of incivility will affect the influence of comments that reveal a slant inconsistent with social media user’ prior attitudes on their behavioral intention to wear masks, but it will not affect the influence of comments that reveal a slant consistent with their preexisting attitudes.
- Hypothesis 5b: the impact of the interaction of comment slant, comment tone, and prior attitudes on social media users’ intentions to wear masks will be mediated by their perception of the influence of the mask-promoting post on others.

In summary, this study aims to investigate how social media users’ polarized attitudes toward mask-wearing and their exposure to a mask-promoting post synchronized with user comments, independently or collectively, affect their compliance with mask-wearing.

## Methods

### Experimental Design

The study used a web-based between-subjects survey experiment with a 2 (comment slant: pro–mask-wearing vs anti–mask-wearing) × 2 (comment tone: civil vs uncivil) design. Participants were recruited from Amazon Mechanical Turk (MTurk), a crowdsourcing platform that allows individuals to outsource tasks, including web-based experiment participation, to registered workers [51]. There has been a long methodological discussion about the data quality obtained from MTurk. While some studies criticize the quality of data obtained from this platform, some indicate that MTurk is a feasible platform for online data collection, especially when strict criteria are applied [52,53]. Therefore, to ensure the quality of our data, we established specific criteria for participant selection (ie, the number of the participants’ approved assignments was >5000, the participants’ approval rating was >95%, and the participants were in the United States). In addition, we incorporated 2 attention checks (ie, select a specific word from the given options). The participation was immediately terminated when participants failed to pass attention checks.

### Ethical Considerations

Participants were recruited from September 29 to October 1, 2020. The study was reviewed and approved by the Human Subjects Ethics Subcommittee of the City University of Hong Kong (2020-55359071) before data collection. In the recruitment announcement posted on MTurk, we informed participants that (1) this study examined their knowledge of and attitudes toward mask-wearing; (2) the participation was fully anonymous, and their self-reported data would be kept confidential; and (3) they could leave the study any time if they wanted. After each participant clicked to agree to a written consent form, which again highlighted these ethical considerations, they continued to participate in the survey. Informed consent was obtained from all participants.

### Stimuli

A mask-promoting post was created and embedded in a fictitious health organization’s Facebook page, as Facebook is widely used by health organizations to promote health initiatives. The post was created based on the guidelines about mask-wearing posted on the official website of the Centers for Disease Control and Prevention in the United States to ensure external validity [54,55]. It was developed using the standard format of fear appeal commonly used in health communication campaigns. To prevent the post from being perceived as an unintended threat to individuals’ freedom, which could undermine the campaign’s effectiveness, we framed it as a low-threat fear appeal by using mild and polite language to recommend mask-wearing [56]. The content and layouts of the post were kept identical across all conditions.

Prior research indicates that exposure to >4 comments does not increase the effect of comment tone [57]. Therefore, we encapsulated 4 comments below the post for each condition. Comment slant was initially created based on actual Facebook users’ expressions on mask-wearing. Across the 2 conditions of comment slant, we matched 2 comments, 1 in each condition, that focused on the same aspects of mask-wearing but expressed opposite opinions and also maintained similar levels of lengths, expression style, and argument strength of the comments. We repeated this procedure for the other comments. This allowed us to generate civil pro–mask-wearing and anti–mask-wearing comments without incurring confounding factors.

Comment tone was manipulated by following the definition of incivility by Coe et al [5]. We added incivility to the previously created comments to derive uncivil pro–mask-wearing and anti–mask-wearing comments. The post and examples of comments used as stimuli are presented in Multimedia Appendix 1.

### Experimental Procedure

The experiment was conducted using the web-based survey software Qualtrics. Before fielding the questionnaire, the survey, including the stimuli and measures, was proofread by 3 native speakers to ensure readability and validity. The technical functionality of the survey platforms and settings was tested by 5 student assistants. The number of items per page and the total pages of the questionnaire distributed were adjusted by Qualtrics based on the devices each participant used, thereby resulting in variations among participants.

All eligible participants could access the survey link posted on the MTurk assignment page. After providing consent for participation, participants were first asked to report their prior attitudes toward mask-wearing, social media use frequency, and mask-wearing practices. The randomizer of Qualtrics enabled us to randomly assign each participant to 1 of the 4 experimental conditions. After being exposed to the stimuli, participants were asked to indicate their responses to the variables of interest, provide demographic information, and answer manipulation check questions. Participants were allowed to review and change their answers using a “back” button at any time before submitting their responses.

The question regarding participants’ prior attitudes toward masks served as a screening item. Participants were asked to rate the extent to which wearing a mask in public during the COVID-19 pandemic was favorable or unfavorable on a 7-point scale (1=very unfavorable, 4=neither unfavorable nor favorable, and 7=very favorable). Participants were categorized as antimaskers (ie, scores <4) and promaskers (ie, scores >4). As this study focused on the effects of polarized attitudes on presumed influence and compliance behavior, participants with neutral attitudes (ie, scores=4) were directed to the end of the survey.

### Participants

A total of 1501 participants provided consent and started the survey, with 522 (34.78%) participants completing all the questions and being included in the final analysis. The view rate was 84.01% (1503/1789), the participation rate was 99.53% (1496/1503), and the completion rate was 34.78% (522/1501). Upon completion of the study, each participant received a debriefing and an incentive of US $0.72. As each worker on MTurk has a unique ID, a unique visitor is defined by the unique ID, rather than cookies used. We also checked IP addresses to ensure that each participant was a unique site visitor. The survey, as an MTurk task, was displayed only once to each participant to avoid repeated registrations. The CONSORT-EHEALTH (Consolidated Standards of Reporting Trials of Electronic and Mobile Health Applications and Online Telehealth) form [58] and the Checklist for Reporting Results of Internet e-Surveys (CHERRIES) form [59] are presented in Multimedia Appendices 2 and 3, respectively, for further clarity.

Participants in the final sample were aged 21 to 77 (mean 41.58, SD 12.35) years. More than half of them were men (291/522, 55.7%). Of the 522 participants, 246 (47.1%) had completed college as their highest level of education, and 416 (79.7%) identified themselves as White. Most participants (317/522, 60.8%) reported that their annual family income ranged from US$20,000 to $74,999. In terms of political identification, 41.5% (217/522) of the participants identified themselves as Democrats, followed by 40.2% (210/522) as Republicans and 18.2% (95/522) as neither Republicans nor Democrats.

### Measures

The measure of presumed influence was adapted from a previous study [60]. Participants were asked to indicate the extent to which they agreed that the social media post of mask promoting had made other people support mask-wearing in public during the COVID-19 pandemic, using a 7-point scale (1=strongly disagree and 7=strongly agree; mean 4.43, SD 2.00). They were also asked to evaluate whether the post had negatively or positively affected others’ attitudes toward mask-wearing, using a 7-point scale (1=in a very negative manner and 7=in a very positive manner; mean 4.20, SD 1.93). These 2 items were highly correlated and were averaged to form the measure of presumed influence (*r*=0.77; *P*<.001; mean 4.32, SD 1.85).

We measured participants’ behavioral intention to wear masks as compliance with health campaigns by adapting the measure used by Dillard and Shen [56]. Participants were asked to estimate the likelihood that they would wear a mask in public in the next week using a 7-point scale, ranging from 1=definitely will not to 7=definitely will (mean 5.61, SD 1.90).

Before being exposed to the experimental stimuli, participants were asked to report their social media use frequency and mask-wearing practices in the last week. Responses to the 2 questions were rated on a 5-point scale, where 1 meant never and 5 meant nearly always (social media use frequency: mean 3.79, SD 0.93 and mask-wearing practices: mean 3.29, SD 1.04).

### Preliminary Statistical Analyses

For randomization checks, a series of 1-way ANOVA were conducted to test the differences in continuous variables, and several chi-square analyses were conducted to test the differences in categorical variables across conditions. In addition, participants were categorized into 2 groups (ie, antimaskers and promaskers) based on the screening question. The 1-way ANOVA and chi-square analyses were repeated to test the differences in demographic variables between antimaskers and promaskers.

### Statistical Analyses for Manipulation Checks

After exposure to experimental materials, participants were asked to report whether they had read the comments below the post. Next, participants were asked to indicate the extent to which they thought the comments were favorable to the post using a 7-point scale (1=very unfavorable and 7=very favorable). We used an independent-samples 2-tailed *t* test to check the difference in perceived slant of comment between participants in the pro–mask-wearing comments condition and those in the anti–mask-wearing comments condition. Descriptive information (ie, mean and SD), *t* value, dfs, and *P* value were reported to illustrate the difference. Then, 1-sample *t* tests were conducted to indicate whether participants’ perceived slant of comment significantly deviated from the midpoint of the scale (ie, 4). We reported *t* value, dfs, and *P* value to indicate the difference*.*

Furthermore, participants were asked to rate the degree of comment incivility using a 7-point scale (1=very uncivil and 7=very civil). We used independent-samples *t* tests to check the difference in perceived civility of comments between participants in the civil comments condition and those in the uncivil comments condition. Descriptive information (ie, mean and SD), *t* value, dfs, and *P* value were reported to illustrate the difference. Then, 1-sample *t* tests were conducted to indicate whether participants’ perceived slants of comment significantly deviated from the midpoint of the scale (ie, 4). We reported *t* value, *df*s, and *P* value to indicate the difference*.*

### Statistical Analyses for Hypotheses Testing

To test the proposed hypotheses concurrently, we used the PROCESS macro (model 12). The PROCESS macro is a regression path analysis modeling tool used to conduct mediation, moderation, and conditional process analysis; it is widely applied in the fields of social, business, and health sciences [61]. Its model 12 tests moderated mediation models. In this study, behavioral intention to wear masks was included as the dependent variable. Prior attitude (0=anti–mask-wearing and 1=pro–mask-wearing) was entered as the independent variable, and comment slant (0=anti–mask-wearing and 1=pro–mask-wearing) and comment tone (0=uncivil and 1=civil) were included as moderators. Participants’ demographics (ie, age, gender, education, income, race, and political identification), mask-wearing frequency, and social media use frequency were included as covariates. Missing values were replaced by mean scores. We reported the unstandardized coefficient (B), unstandardized SE, *P* value, and 95% CI, which indicate the effects of participants’ prior attitudes, comments slant, comment tone, and presumed influence on their intention to wear masks. In addition, the effect size, SE, and 95% CI were reported to show the conditional direct and indirect effects of comment slant, comment tone, and prior attitudes on behavioral intention.

### Statistical Analyses for Sensitivity Analysis

Two sensitivity analyses were conducted. First, we calculated attitude extremity by subtracting 4 from the value chosen by promaskers in the screening question and by subtracting the value chosen by antimaskers from 4 (ie, 1=low extremity, 2=medium extremity, and 3=high extremity). We controlled for this variable in sensitivity analysis 1. Second, we added the variables stepwise to the regression models—main effects first and then the interaction terms—to better demonstrate the main effects in sensitivity analysis 2.

## Results

### Preliminary Analyses

**Figure 1:**
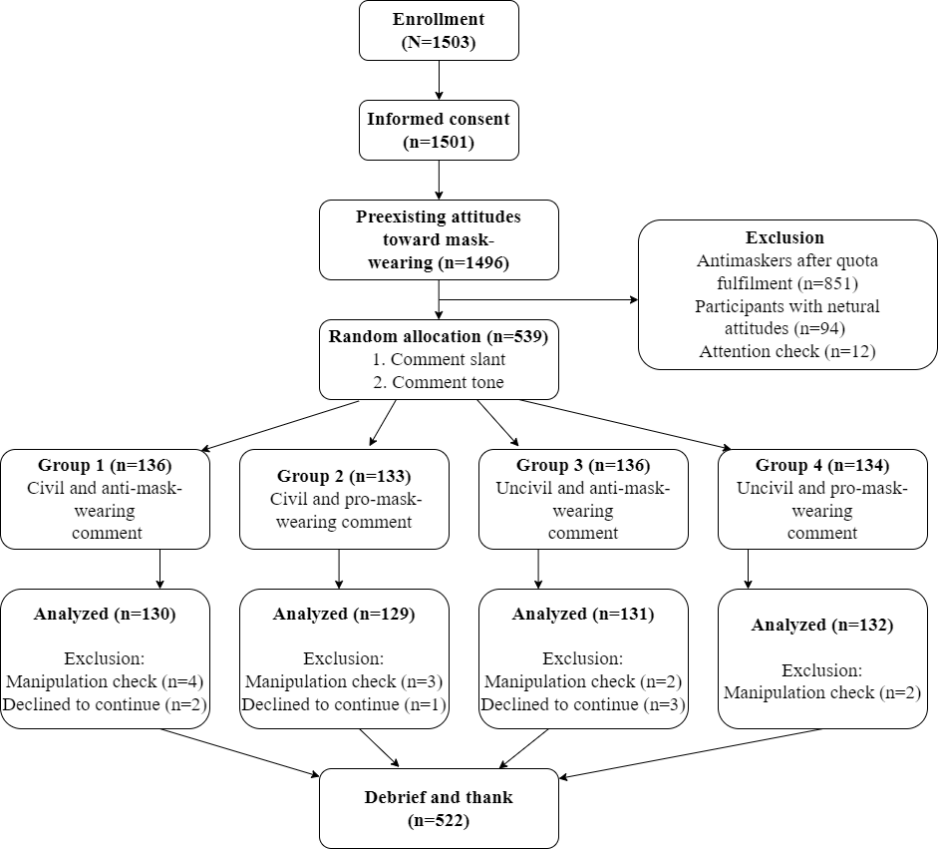
Participation flowchart.

A CONSORT (Consolidated Standards of Reporting Trials) flow diagram for the participants is presented in Figure 1. The demographic information across groups is presented in Multimedia Appendix 4. A series of 1-way ANOVAs indicated that there were no significant differences in participants’ age (*P*=.77), education (*P*=.37), and annual income (*P*=.54) across conditions. Chi-square analyses also showed no significant differences in participants’ gender (*P*=.42), race (*P*=.97), and political identification (*P*=.21) across conditions. In addition, among 522 participants, 269 (51.5%) had unfavorable attitudes toward mask-wearing (ie, antimaskers), whereas 253 (48.5%) held favorable attitudes toward mask-wearing (ie, promaskers). No significant differences in age (*P*=.91), gender (*P*=.91), and annual income (*P*=.51) were found between antimaskers and promaskers. However, promaskers (mean 5.62, SD 1.04) reported higher levels of education than antimaskers (mean 5.32, SD 1.19; t_517.16_=3.05; *P*=.002). Therefore, basic demographic factors were controlled in later analysis to adjust for the differences in the sample.

### Manipulation Checks

Those who reported not reading comments were excluded (11/539, 2%). Participants in the pro–mask-wearing comments condition considered the comments to be more favorable to the post (mean 5.50, SD 1.85) than those in the anti–mask-wearing comments condition (mean 1.70, SD 1.51; t_499.56_=25.75; *P*<.001). One-sample *t* tests indicated that both participants exposed to pro–mask-wearing comments (t_260_=13.10; *P*<.001) or anti–mask-wearing comments (t_260_=24.68; *P*<.001) perceived the comments to significantly deviate from the midpoint of the scale (ie, 4). Next, participants in the civil comments condition considered the comments more civil (mean 4.28, SD 1.88) than those in the uncivil condition (mean 1.92, SD 1.56; t_500.29_=15.53; *P*<.001). One-sample *t* tests showed that both participants exposed to civil comments (t_258_=2.38; *P*=.02) and uncivil comments (t_262_=21.53; *P*<.001) perceived the comment tone to significantly deviate from the midpoint of 4.

### Hypotheses Testing

The results of hypotheses testing for the separate and combined effects of comment slant, comment tone, and prior attitudes on presumed influence and mask-wearing intention are reported in Table 1.

**Table 1:** The influence of comment slant, comment tone, and prior attitudes on presumed influence and behavioral intention to wear masks^a^.

	Presumed influence			Mask-wearing intention	
	B (SE; 95% CI)	*P* value	B (SE; 95% CI)		*P* value
Age	–0.00 (0.01; –0.01 to 0.01)	.81	0.01 (0.00; –0.00 to 0.01)		.19
Gender	0.03 (0.13; –0.23 to 0.30)	.81	0.16 (0.10; –0.03 to 0.35)		.09
Education	0.13 (0.06; 0.01 to 0.25)	.04	–0.09 (0.05; –0.18 to –0.01)		.04
Income	–0.11 (0.04; –0.18 to –0.04)	.003	0.02 (0.03; –0.03 to 0.08)		.40
Race	–0.09 (0.17; –0.42 to 0.24)	.59	0.20 (0.12; –0.04 to 0.44)		.10
Republican	0.15 (0.19; –0.23 to 0.53)	.43	0.02 (0.14; –0.26 to 0.29)		.91
Democrat	0.52 (0.20; 0.14 to 0.90)	.008	0.03 (0.14; –0.25 to 0.31)		.82
Mask-wearing frequency	0.22 (0.06; 0.09 to 0.35)	.001	1.08 (0.05; 0.99 to 1.71)		<.001
Social media use frequency	0.13 (0.07; –0.02 to –0.27)	.08	0.01 (0.05; –0.09 to 0.11)		.87
Prior attitude	0.19 (0.28; –0.37 to 0.74)	.51	0.86 (0.20; 0.45 to 1.26)		<.001
Comment slant	1.49 (0.26; 0.98 to 2.00)	<.001	–0.06 (0.19; –0.44 to 0.32)		.74
Comment tone	0.63 (0.26; 0.12 to 1.14)	.02	–0.44 (0.19; –0.81 to –0.07)		.02
Prior attitude × comment slant	0.27 (0.37; –0.46 to 1.00)	.47	–0.15 (0.27; –0.68 to 0.38)		.58
Prior attitude × comment tone	–0.26 (0.37; –1.00 to 0.47)	.48	0.34 (0.27; –0.19 to 0.87)		.21
Comment slant × comment tone	0.09 (0.36; –0.62 to 0.81)	.80	0.79 (0.26; 0.28 to 1.31)		.003
Prior attitude × comment slant × comment tone	0.55 (0.53; –0.48 to 1.59)	.30	–0.84 (0.38; –1.59 to –0.09)		.03
Presumed influence	—^b^	—	0.07 (0.03; 0.01 to 0.13)		.03

^a^Model summary: presumed influence, *F*_16,505_=18.99; mask-wearing intention, *F*_17,504_=67.01.

^b^Not applicable (at this stage, the presumed influence is the dependent variable).

The results of bootstrapping for the conditional direct and indirect effects of comment slant, comment tone, and prior attitudes on behavioral intention to wear masks are summarized in Table 2.

**Table 2:** The conditional direct and indirect effects of comment slant, comment tone, and prior attitudes on behavioral intention.

			Indirect effects (mediator: presumed influence), effect size (SE; 95% CI)		Direct effects, effect size (SE ; 95% CI)
**Comment slant**					
	Uncivil × antimaskers	0.10 (0.05; 0.01 to 0.21)		–0.06 (0.19; –0.44 to 0.32)	
	Uncivil × promaskers	0.12 (0.06; 0.01 to 0.25)		–0.21 (0.20; –0.60 to 0.18)	
	Civil × antimaskers	0.11 (0.06; 0.01 to 0.23)		0.73 (0.19; 0.35 to 1.10)	
	Civil × promaskers	0.16 (0.08; 0.01 to 0.33)		–0.26 (0.21; –0.67 to 0.15)	
**Comment tone**					
	Anti–mask-wearing × antimaskers	0.04 (0.03; –0.00 to 0.11)		–0.44 (0.19; –0.81 to –0.07)	
	Anti–mask-wearing × promaskers	0.03 (0.03; –0.02 to 0.09)		–0.10 (0.20; –0.48 to 0.28)	
	Pro–mask-wearing × antimaskers	0.05 (0.03; 0.001 to 0.120)		0.35 (0.19; –0.02 to 0.72)	
	Pro–mask-wearing × promaskers	0.07 (0.04; 0.003 to 0.149)		–0.15 (0.19; –0.53 to 0.23)	
**Prior attitudes**					
	Anti–mask-wearing × uncivil	0.01 (0.03; –0.04 to 0.07)		0.86 (0.20; 0.45 to 1.26)	
	Anti–mask-wearing × civil	–0.01 (0.02; –0.05 to 0.04)		1.20 (0.20; 0.79 to 1.60)	
	Pro–mask-wearing × uncivil	0.03 (0.03; –0.01 to 0.09)		0.71 (0.20; 0.32 to 1.10)	
	Pro–mask-wearing × civil	0.05 (0.03; 0.00 to 0.11)		0.21 (0.20; –0.19 to 0.60)	

As for hypotheses 1a and 1b, the regression results in Table 1 showed that there was no significant association between comment slant and behavioral intention (B=–0.06; *P*=.74). Hence, hypothesis 1a was not supported. Nevertheless, we found that compared with anti–mask-wearing comments, pro–mask-wearing comments were found to increase presumed influence (B=1.49; *P*<.001), and this presumed influence was positively associated with participants’ behavioral intention to wear masks (B=0.07; *P*=.03). The bootstrapping results showed that the direct effect of comment slant on behavioral intention was significant only among antimaskers who read civil comments (B=0.73, SE 0.79; 95% CI 0.35-1.10). Comment slant posed an indirect influence on behavioral intention through the mediation of presumed influence, regardless of participants’ prior attitudes or comment tone (Table 2). Hence, hypothesis 1b was supported by the data.

Next, for hypotheses 2a and 2b, results in Table 1 showed that there was a significant but negative association between comment tone and behavioral intention to wear masks (B=–0.44; *P*=.02). Hence, hypothesis 2a was not supported. As for hypothesis 2b, the effect of comment tone on presumed influence was positively significant (B=0.63; *P*=.02), and the association between presumed influence and intention to wear masks was also positively significant (B=0.07; *P*=.03). The bootstrapping results showed that comment tone posed a direct influence on behavioral intention to wear masks only when antimaskers encountered anti–mask-wearing comments (B=–0.44, SE 0.19; 95% CI –0.81 to –0.07). In addition, comment tone posed an influence on behavioral intention via the mediating effects of presumed influence when the comments were pro–mask-wearing, regardless of participants’ prior attitudes (Table 2). Hence, hypothesis 2b was partially supported.

As for hypotheses 3a and 3b, the results in Table 1 showed that the direct effect of prior attitudes on behavioral intention to wear masks was significant (B=0.86; *P*<.001). Hence, hypothesis 3a received support. However, the effect of prior attitudes on presumed influence was not significant (B=0.19; *P*=.51). The bootstrapping results (Table 2) indicated that as long as the comments were uncivil or anti–mask-wearing, participants’ prior attitudes were directly associated with their behavioral intention. Only when comments were pro–mask-wearing and civil, prior attitudes affected behavioral intention through presumed influence. Hence, we mostly could not corroborate hypothesis 3b.

**Figure 2:**
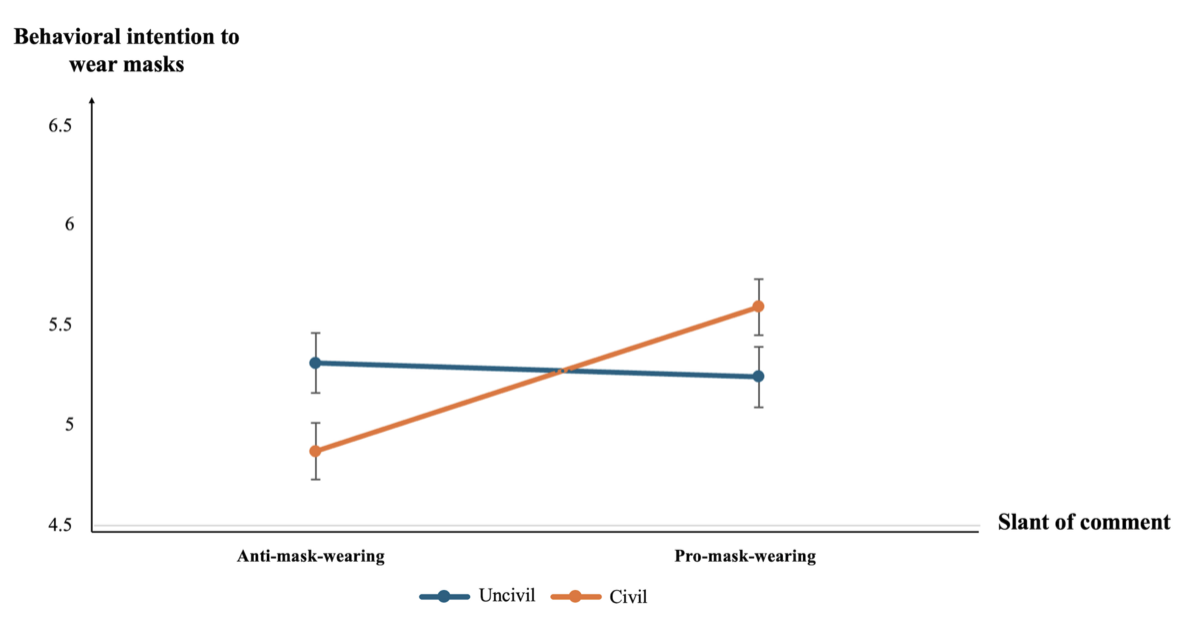
The interaction effect of comment slant and comment tone on the behavioral intention to wear masks.

Regarding hypothesis 4a, Table 1 shows that the interaction had a significant and direct effect on behavioral intention (B=0.79; *P*=.003). As shown in Figure 2, when expressed in a civil way, opposing comments (mean 5.46, SD 0.10) decreased the mask-wearing intention than supporting comments (mean 5.70, SD 0.10); while when expressed in an uncivil way, the effects of comments slant was reversed such that supporting comments (mean 5.59, SD 0.10) decreased the mask-wearing intention, compared with opposing comments (mean 5.73, SD 0.10). Hence, hypothesis 4a was supported. For hypothesis 4b, the results showed that the interaction between comment slant and comment tone did not significantly predict presumed influence (B=0.09; *P*=.80). Hence, hypothesis 4b was not supported.

**Figure 3:**
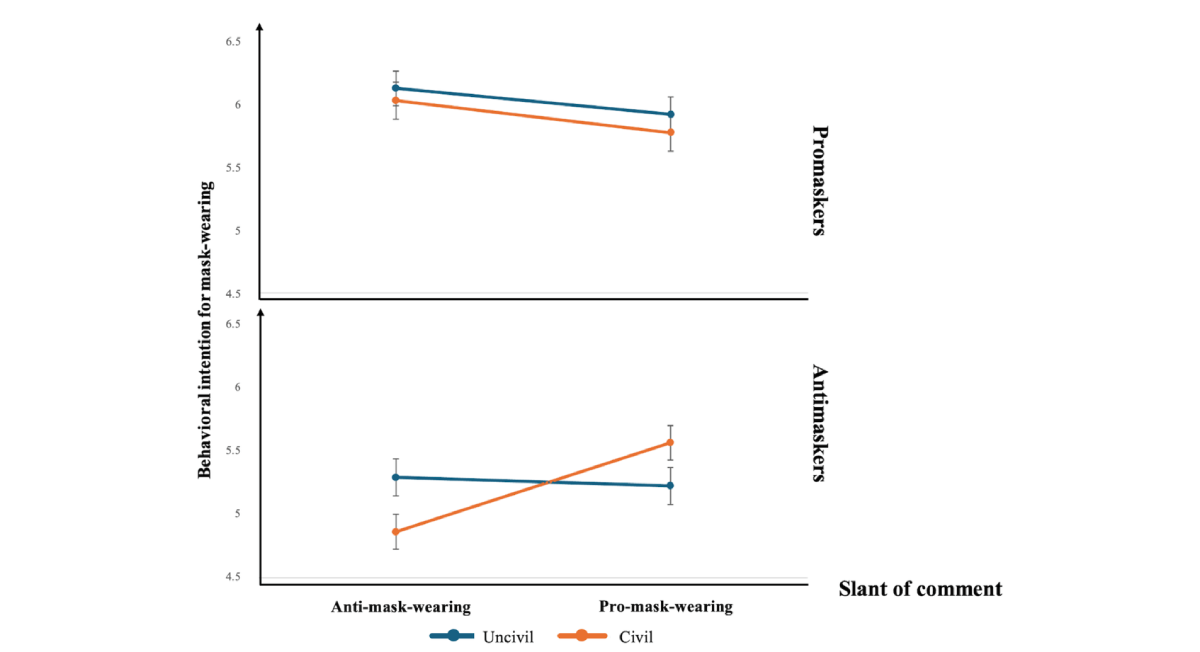
The interaction effect of comment slant, comment tone, and prior attitudes on the behavioral intention to wear masks.

For hypotheses 5a and 5b, the results (Table 1) showed that the interaction was significant for behavioral intention (B=–0.84; *P*=.03) but not for presumed influence (B=0.55; *P*=.30). However, the effect of the interaction on behavioral intention was different from what we expected. As shown in Figure 3, for promaskers, behavioral intention to wear masks remained similar when they saw uncivil comments or civil comments, regardless of whether the comments were anti–mask-wearing (civil: mean 6.06, SD 0.15 and uncivil: mean 6.16, SD 0.14; *P*=.61) or pro–mask-wearing (civil: mean 5.80, SD 0.15 and uncivil: mean 5.95, SD 0.14; *P*=.45). In contrast, among antimaskers, their behavioral intention remained similar when they viewed uncivil pro–mask-wearing comments (mean 5.24, SD 0.13) and civil pro–mask-wearing comments (mean 5.59, SD 0.14; *P*=.06). Nevertheless, their behavioral intention was stronger when they read uncivil anti–mask-wearing comments (mean 5.31, SD 0.15) compared to civil anti–mask-wearing comments (mean 4.87, SD 0.14; *P*=.02). Hence, hypothesis 5a was partially supported, but hypothesis 5b was not supported.

### Sensitivity Analyses

Results from the sensitivity analyses, which included attitude extremity as an additional control variable and involved applying stepwise multiple linear regression (Multimedia Appendices 5-8) were consistent with the main results.

## Discussion

### Principal Findings

This study investigated how polarized and hostile user comments below a health campaign message on social media and social media users’ polarized attitudes concurrently affected their perception of the campaign’s influence on others and their compliance with the promoted health behavior. Results showed that compared with anti–mask-wearing comments, pro–mask-wearing comments enhanced presumed influence and health compliance of mask-wearing, but incivility in the comments hindered the positive impact of pro–mask-wearing comments. Antimaskers demonstrated increased compliance when they were unable to find civil support for their opinion in the social media environment. The summary of the research hypotheses and corresponding results are presented in Table 3.

**Table 3:** Summary of hypotheses and results.

Hypotheses	Results
Hypothesis 1a: social media users will have weaker intentions to wear masks when exposed to anti–mask-wearing comments below a mask-promoting post than when exposed to pro–mask-wearing comments.	Not supported
Hypothesis 1b: the association between comment slant and intentions to wear masks will be mediated by social media users’ perception of the influence of the mask-promoting post on others.	Supported
Hypothesis 2a: social media users will have weaker intentions to wear masks when exposed to uncivil comments below a mask-promoting post than when exposed to civil comments.	Not supported
Hypothesis 2b: the association between comment tone and intentions to wear masks will be mediated by social media users’ perception of the influence of the mask-promoting post on others.	Supported only when the mask-promoting post was accompanied by pro–mask-wearing comment
Hypothesis 3a: social media users will have weaker intentions to wear masks when they have unfavorable attitudes toward mask-wearing than when they have favorable attitudes.	Supported
Hypothesis 3b: the association between prior attitudes toward mask-wearing and intentions to wear masks will be mediated by social media users’ perception of the influence of the mask-promoting post on others.	Supported only when the mask-promoting post was accompanied by pro–mask-wearing and civil comment
Hypothesis 4a: comment tone will moderate the effect of comment slant on social media users’ intentions to wear masks, such that the effect of comment slant on behavioral intention will be stronger when comments are expressed in a civil manner compared to in an uncivil manner.	Supported
Hypothesis 4b: the interaction effect of comment slant and comment tone on social media users’ intentions to wear masks will be mediated by their perception of the influence of the mask-promoting post on others.	Not supported
Hypothesis 5a: there is an interaction among comment slant, comment tone, and prior attitudes on social media users’ intentions to wear masks, such that the influence of incivility will affect the influence of comments that reveal a slant inconsistent with social media user’ prior attitudes on their behavioral intention to wear masks, but it will not affect the influence of comments that reveal a slant consistent with their preexisting attitudes.	Supported only among antimaskers
Hypothesis 5b: the impact of the interaction of comment slant, comment tone, and prior attitudes on social media users’ intentions to wear masks will be mediated by their perception of the influence of the mask-promoting post on others.	Not supported

### Results and Comparison With Prior Work

First, comment slant remained a cornerstone driving individuals’ presumed influence of the mask-promoting post and their compliance with mask-wearing. Compared to pro–mask-wearing comments, anti–mask-wearing comments always reduced participants’ presumed influence of mask-wearing posts, which further weakened their behavioral intention to wear masks, regardless of comment tone and their prior attitudes toward mask-wearing. These findings suggest that comments can serve as a source of misleading information. Although attitudinal consensus is inferred from the comments left by anonymous and limited others, these comments may lead social media users to develop inaccurate beliefs that the comments reflect public opinion from people in general. These beliefs may influence their health-related compliance behaviors.

In addition, incivility affected the presumed influence of a health message, but only when the comments below the message expressed supportive opinions. It is likely that pro–mask-wearing comments below the health message signal the presumed influence of the message on others’ acceptance, and incivility acts as a negative cue that hinders the exemplification effect and indicates that the highly homogeneous and consistent opinion environment depicted in the comments may not be accurate. In contrast, anti–mask-wearing comments have explicitly represented others’ resistance to the main health message, and the presence of incivility only signals a similar cue of others’ resistance.

These effects of comment slant and comment tone advance the other-consciousness perspective of the IPI process in the context of digital health campaigns. Individuals’ perception of others’ reception of a media message is influenced by affordances offered by social media. Even when information on source credibility or audience size is absent, the presumed influence of social media messages still adjusts individuals’ compliance behavior accordingly. Social media users take the roles of both content producers and commenters. The opinion environment is highly prone to produce and spread misleading information due to the lack of professional gatekeepers and polarized opinion climate. The IPI model demonstrates a psychological process through which individuals’ exposure to health information and relevant discussions on social media affects their compliance with promoted health behaviors. Therefore, it is crucial to consider both the direct and indirect effects of social media comments below health-persuasive messages on public health outcomes when examining the persuasiveness of digital health communication.

With concrete clues about others’ reactions to health persuasion obtained from comments, individuals no longer rely solely on their prior attitudes to infer the influence of a health campaign on others. These findings somewhat challenge the self-centric perspective of IPI. This change can be explained by the evolving media landscape. Previous studies support the self-centric perspective of IPI in the context of traditional media, where traditional media audiences have limited access to others’ reactions to a message and are compelled to rely on their prior attitudes for inference. In social media, users can directly see others’ reactions to a message. They no longer need to fully rely on personal attitudes to infer media influence on others. Comments serve as crucial sources for them to infer the influence of social media posts on others.

Only when approving and civil comments are present, prior attitudes can affect behavioral intentions through individuals’ presumed media influence on others. One possible explanation is that individuals in general are subject to negative bias, that is, they are particularly susceptible to information that contains negativity or risks. Anti–mask-wearing comments or incivility impressed and influenced participants because these comments might exaggerate the negative side of mask-wearing and demonstrate hostility among commenters. Therefore, individuals’ perception of others’ reactions to the main message is believed to be influenced by negative cues rather than prior attitudes. Civil pro–mask-wearing comments suggested no cues of negativity, and individuals then relied on their prior attitudes to infer the perception of the post’s influence on others.

In most cases, favorable prior attitudes toward mask-wearing directly enhance individuals’ behavioral intention compared to unfavorable prior attitudes. Nevertheless, the influence of prior attitudes on behavioral intentions can be altered by social media comments ensuing digital health communication. Specifically, civil pro–mask-wearing comments directly enhance antimaskers’ behavioral intention to wear masks more than uncivil pro–mask-wearing comments, whereas uncivil anti–mask-wearing comments turn out to enhance antimaskers’ behavioral intention to wear masks more than civil anti–mask-wearing comments. An explanation is that individuals may psychologically dissociate themselves from a group whose members belong to a relatively inferior group [62]; incivility is seen as impolite and undesirable, and individuals may avoid belonging to a group whose members are rude and uncivil. The findings indicate that individuals engage in biased information processing only when they find civil support for their prior opinions, regardless of whether the support is narrated in the main message or in the comments.

Relatedly, while we suspected that antimask attitudes differing from the post advocacy would be associated with less presumed influence, there is a possibility that opponents of mask-wearing may adhere to conspiracy theories. Such individuals might suspect that everyone around them has been brainwashed by governmental health campaigns, thereby leading to very high presumed influence. In other words, there might be a curvilinear relationship between prior attitudes and presumed influence or a linear relationship between attitude strength and presumed influence among antimaskers. Therefore, we conducted additional tests and found that these possibilities were not supported by our data. These findings suggest that in the era of new media, where user responses to health campaigns are publicly visible, judgments about the presumed influence of a post rely more on these visible examples than on personal prior attitudes.

### Limitations and Future Directions

This study has several limitations that should be acknowledged. First, we edited the comments to maintain consistent argument strength across conditions, and therefore, the level of perceived authenticity in the comments may differ. Furthermore, we used default Facebook avatars in the experimental stimuli. Uncivil social media comments coupled with default avatars may be regarded as bot accounts, given the heavily politicized discussion on mask-wearing in the United States. The perception of commenters as bots may affect the presumed influence accordingly. These two aspects suggest that the perceived unrealism of the stimuli, particularly the user comments created in this study, may reduce the validity of the findings. Given that, future studies would benefit from measuring the perceived realism of comments and controlling it as a covariate in the analyses.

Second, this study focuses on the effects of user comments and prior attitudes, leaving the main effectiveness of the health campaign post unexamined. Likewise, the interaction effects between the post and its accompanying comments on polarized publics’ presumed influence and behavioral intentions remain unexplored. The combined effects of comment slant and comment tone may vary depending on the post presented together with the comments. The lack of examination of the interplay between comments and the post may hinder a nuanced understanding of the combined effects of digital information. Future studies are encouraged to consider the effectiveness of a post and its interaction with comments.

Third, participants were required to read the post and accompanying comments, which may not reflect real-life scenarios where individuals may choose whether to browse the information or not. Participants may generate bias through the procedure of providing informed consent and reading the survey questions, influencing their later answers. These factors could also affect the validity of the study findings. Future research should use experimental designs that better reflect real-world settings.

Fourth, although the IPI model has long been used in health communication research and is valuable for addressing specific questions in this study, it primarily focuses on the indirect effects of health campaigns. However, within the context of public health communication, there are various alternative theoretical explanations for the effectiveness or ineffectiveness of health campaigns. For instance, fear appeals suggest that how information is presented by the supply side of communication can influence individuals’ emotional reactions and health behavior changes [63]. Psychological reactance can be another relevant concept with regard to campaign failure from the recipients’ perspective. When individuals perceive health campaigns to threaten their behavioral freedom, they react in ways contrary to the campaign’s intent, resulting in communication failure [56]. In other words, the findings from this study should be interpreted as 1 aspect of evaluating the effectiveness of health campaigns. To gain a comprehensive understanding of their effectiveness, these findings should be integrated with insights from other theoretical perspectives.

### Conclusions and Implications

Despite these limitations, our study suggests that online health campaigns may yield desirable outcomes when civil and supportive comments are present. Moreover, social media users often engage in biased processing of health persuasion and rely heavily on their prior attitudes to guide their subsequent compliance behaviors. Unfavorable prior attitudes toward health behaviors can harm the effects of digital health communication only when individuals find civil and consistent evidence supporting their unfavorable opinions. Therefore, it is beneficial to encourage social media users to leave civil and supportive comments on digital health campaigns. In addition, misinformation and incivility in online comment sections should be moderated by relevant media platforms. Moreover, relevant information literacy programs should be delivered to the public to prevent them from being misled by biased user comments. Theoretically, this study explores the other-consciousness and self-centered perspectives of presumed influence in the context of social media health campaigns, where messages are presented together with extensive polarized and hostile user comments. People rely on online commentary and their prior attitudes to infer the presumed influence of health campaigns.

## Appendix

Multimedia Appendix 1 Screenshots of the experimental stimuli.

Multimedia Appendix 2 Checklist for reporting of web-based and mobile randomized controlled trials (CONSORT-EHEALTH [Consolidated Standards of Reporting Trials of Electronic and Mobile Health Applications and Online Telehealth]).

Multimedia Appendix 3 Checklist for Reporting Results of Internet e-Surveys (CHERRIES).

Multimedia Appendix 4 Participants’ demographics across groups.

Multimedia Appendix 5 Sensitivity analysis 1 (regression results).

Multimedia Appendix 6 Sensitivity analysis 1 (direct and indirect effects tests).

Multimedia Appendix 7 Sensitivity analysis 2 (dependent variable: presumed influence).

Multimedia Appendix 8 Sensitivity analysis 2 (dependent variable: mask-wearing intention).

## Abbreviations

CONSORT: Consolidated Standards of Reporting Trials
CONSORT-EHEALTH: Consolidated Standards of Reporting Trials of Electronic and Mobile Health Applications and Online Telehealth
IPI: influence of presumed influence
MTurk: Mechanical Turk

## Data Availability

The datasets generated and analyzed during this study are available from the corresponding author on reasonable request.

## References

[1] Rao A, Morstatter F, Hu M, Chen E, Burghardt K, Ferrara E, Lerman K (2021). Political partisanship and antiscience attitudes in online discussions about COVID-19: Twitter content analysis. J Med Internet Res.

[2] Oksanen A, Garcia D, Sirola A, Näsi M, Kaakinen M, Keipi T, Räsänen P (2015). Pro-anorexia and anti-pro-anorexia videos on YouTube: sentiment analysis of user responses. J Med Internet Res.

[3] Lenti J, Mejova Y, Kalimeri K, Panisson A, Paolotti D, Tizzani M, Starnini M (2023). Global misinformation spillovers in the vaccination debate before and during the COVID-19 pandemic: multilingual Twitter study. JMIR Infodemiology.

[4] Stevens H, Rasul ME, Oh YJ (2022). Emotions and incivility in vaccine mandate discourse: natural language processing insights. JMIR Infodemiology.

[5] Coe K, Kenski K, Rains SA (2014). Online and uncivil? Patterns and determinants of incivility in newspaper website comments. J Commun.

[6] Kite J, Chan L, MacKay K, Corbett L, Reyes-Marcelino G, Nguyen B, Bellew W, Freeman B (2023). A model of social media effects in public health communication campaigns: systematic review. J Med Internet Res.

[7] Lu F, Sun Y (2022). COVID-19 vaccine hesitancy: the effects of combining direct and indirect online opinion cues on psychological reactance to health campaigns. Comput Human Behav.

[8] Lu F, Sun Y, Oktavianus J (2023). Resistance to masks during the COVID-19 pandemic: how user comments drive psychological reactance to health campaigns. Health Commun.

[9] Sun Y, Lu F (2023). How misinformation and rebuttals in online comments affect people's intention to receive COVID-19 vaccines: the roles of psychological reactance and misperceptions. Journal Mass Commun Q.

[10] Wang S (2020). The influence of anonymity and incivility on perceptions of user comments on news websites. Mass Commun Soc.

[11] Lee FL, Liang H, Tang GK (2019). Online incivility, cyberbalkanization, and the dynamics of opinion polarization during and after a mass protest event. Int J Commun.

[12] Kim Y, Kim Y (2019). Incivility on Facebook and political polarization: the mediating role of seeking further comments and negative emotion. Comput Human Behav.

[13] Gunther AC, Storey JD (2003). The influence of presumed influence. J Commun.

[14] Chung JE (2018). Peer influence of online comments in newspapers: applying social norms and the social identification model of deindividuation effects (SIDE). Soc Sci Comput Rev.

[15] Cho H, Shen L, Peng L (2021). Examining and extending the influence of presumed influence hypothesis in social media. Media Psychol.

[16] Gunther AC, Bolt D, Borzekowski DL, Liebhart JL, Dillard JP (2006). Presumed influence on peer norms: how mass media indirectly affect adolescent smoking. J Commun.

[17] Yoo W, Yang J, Cho E (2016). How social media influence college students' smoking attitudes and intentions. Comput Human Behav.

[18] Kluck JP, Krämer NC (2022). Appraising uncivil comments in online political discussions: how do preceding incivility and senders’ stance affect the processing of an uncivil comment?. Commun Res.

[19] Pascual-Ferrá P, Alperstein N, Barnett DJ, Rimal RN (2021). Toxicity and verbal aggression on social media: polarized discourse on wearing face masks during the COVID-19 pandemic. Big Data Soc.

[20] Yiannakoulias N, Darlington JC, Slavik CE, Benjamin G (2022). Negative COVID-19 vaccine information on Twitter: content analysis. JMIR Infodemiology.

[21] Majó-Vázquez S, Nielsen R, Verdú J, Rao N, Domenico N, Papaspiliopoulos O (2020). Volume and patterns of toxicity in social media conversations during the COVID-19 pandemic. Reuters Institute for the Study of Journalism.

[22] Block R Jr, Burnham M, Kahn K, Peng R, Seeman J, Seto C (2022). Perceived risk, political polarization, and the willingness to follow COVID-19 mitigation guidelines. Soc Sci Med.

[23] Ho SS, Lee EW, Ng K, Leong GS, Tham TH (2016). For fit's sake: a norms-based approach to healthy behaviors through influence of presumed media influence. Health Commun.

[24] Chia SC, Sun Y, Lu F, Gudmundsdottir A (2023). Doxing, regulation, and privacy protection: expanding the behavioral consequences of the third-person effect. Asian J Commun.

[25] Hong Y, Kim S (2020). Influence of presumed media influence for health prevention: how mass media indirectly promote health prevention behaviors through descriptive norms. Health Commun.

[26] Lin CA, Xu X, Dam L (2020). Information source dependence, presumed media influence, risk knowledge, and vaccination intention. Atl J Commun.

[27] Tu C, Li X (2024). Personal versus societal risk: examining social media influence on individual and collective behaviors for COVID-19 containment. Soc Media Society.

[28] Shi R, Messaris P, Cappella JN (2014). Effects of online comments on smokers' perception of anti-smoking public service announcements. J Comput Mediat Commun.

[29] Tu C, Li X (2022). Social support and reputational defense strategies of Chinese social networking site users in Sun Yang’s controversy. Public Relat Rev.

[30] Winter S, Brückner C, Krämer NC (2015). They came, they liked, they commented: social influence on facebook news channels. Cyberpsychol Behav Soc Netw.

[31] Zillmann D (2006). Exemplification effects in the promotion of safety and health. J Commun.

[32] Lee EJ, Jang YJ (2010). What do others’ reactions to news on internet portal sites tell us? Effects of presentation format and readers’ need for cognition on reality perception. Commun Res.

[33] Waddell TF, Sundar SS (2020). Bandwagon effects in social television: how audience metrics related to size and opinion affect the enjoyment of digital media. Comput Human Behav.

[34] Chia SC, Wen N (2010). College men’s third-person perceptions about idealized body image and consequent behavior. Sex Roles.

[35] Anderson AA, Yeo SK, Brossard D, Scheufele DA, Xenos MA (2018). Toxic talk: how online incivility can undermine perceptions of media. Int J Public Opin Res.

[36] Prochazka F, Weber P, Schweiger W (2016). Effects of civility and reasoning in user comments on perceived journalistic quality. Journal Stud.

[37] Bode L, Vraga EK, Tully M (2020). Do the right thing: tone may not affect correction of misinformation on social media. Harvard Kennedy School (HKS) Misinformation Review.

[38] Masullo GM, Lu S, Fadnis D (2020). Does online incivility cancel out the spiral of silence? A moderated mediation model of willingness to speak out. New Media Soc.

[39] Waddell TF, Bailey A (2017). Inspired by the crowd: the effect of online comments on elevation and universal orientation. Commun Monogr.

[40] Fields JM, Schuman H (1976). Public beliefs about the beliefs of the public. Public Opin Q.

[41] Cherniak C, Nisbett R, Ross L (1983). Human inference: strategies and shortcomings of social judgment. Philos Rev.

[42] Marks G, Miller N (1987). Ten years of research on the false-consensus effect: an empirical and theoretical review. Psychol Bull.

[43] Shen L, Huggins C (2013). Testing the model of influence of presumed influence in a boundary condition: the impact of question order. Hum Commun Res.

[44] Kunda Z (1990). The case for motivated reasoning. Psychol Bull.

[45] Vraga EK, Kim SC, Cook J (2019). Testing logic-based and humor-based corrections for science, health, and political misinformation on social media. J Broadcast Electron Media.

[46] Kim H, Seo Y, Yoon HJ, Han JY, Ko Y (2021). The effects of user comment valence of Facebook health messages on intention to receive the flu vaccine: the role of pre-existing attitude towards the flu vaccine and psychological reactance. Int J Advert.

[47] Georgiou N, Delfabbro P, Balzan R (2020). COVID-19-related conspiracy beliefs and their relationship with perceived stress and pre-existing conspiracy beliefs. Pers Individ Dif.

[48] Ajzen I (1991). The theory of planned behavior. Organ Behav Hum Decis Process.

[49] Pornpitakpan C (2006). The persuasiveness of source credibility: a critical review of five decades' evidence. J Appl Soc Pyschol.

[50] Hogg MA (2001). A social identity theory of leadership. Pers Soc Psychol Rev.

[51] Aguinis H, Villamor I, Ramani RS (2020). MTurk research: review and recommendations. J Manag.

[52] Mortensen K, Hughes TL (2018). Comparing Amazon's mechanical Turk platform to conventional data collection methods in the health and medical research literature. J Gen Intern Med.

[53] Kennedy R, Clifford S, Burleigh T, Waggoner PD, Jewell R, Winter NJ (2020). The shape of and solutions to the MTurk quality crisis. Political Sci Res Methods.

[54] Community respirators and masks. Centers for Disease Control and Prevention.

[55] About COVID-19. Centers for Disease Control and Prevention.

[56] Dillard JP, Shen L (2005). On the nature of reactance and its role in persuasive health communication. Commun Monogr.

[57] Rösner L, Winter S, Krämer NC (2016). Dangerous minds? Effects of uncivil online comments on aggressive cognitions, emotions, and behavior. Comput Human Behav.

[58] Eysenbach G, CONSORT-EHEALTH Group (2011). CONSORT-EHEALTH: improving and standardizing evaluation reports of web-based and mobile health interventions. J Med Internet Res.

[59] Eysenbach G (2004). Improving the quality of web surveys: the Checklist for Reporting Results of Internet E-Surveys (CHERRIES). J Med Internet Res.

[60] Tsfati Y, Cohen J, Gunther AC (2010). The influence of presumed media influence on news about science and scientists. Sci Commun.

[61] Hayes AF (2017). Introduction to Mediation, Moderation, and Conditional Process Analysis: A Regression-Based Approach.

[62] Hornsey MJ (2008). Social identity theory and self‐categorization theory: a historical review. Soc Personal Psychol Compass.

[63] Moussaoui LS, Claxton N, Desrichard O (2021). Fear appeals to promote better health behaviors: an investigation of potential mediators. Health Psychol Behav Med.

