# Supplementary figures and images for: The Impact of Comment Slant and Comment Tone on Digital Health Communication Among Polarized Publics: A Web-Based Survey Experiment
Source: J Med Internet Res. 2024 Nov 15;26:e57967. doi: 10.2196/57967 (PMC11607566; doi:10.2196/57967)

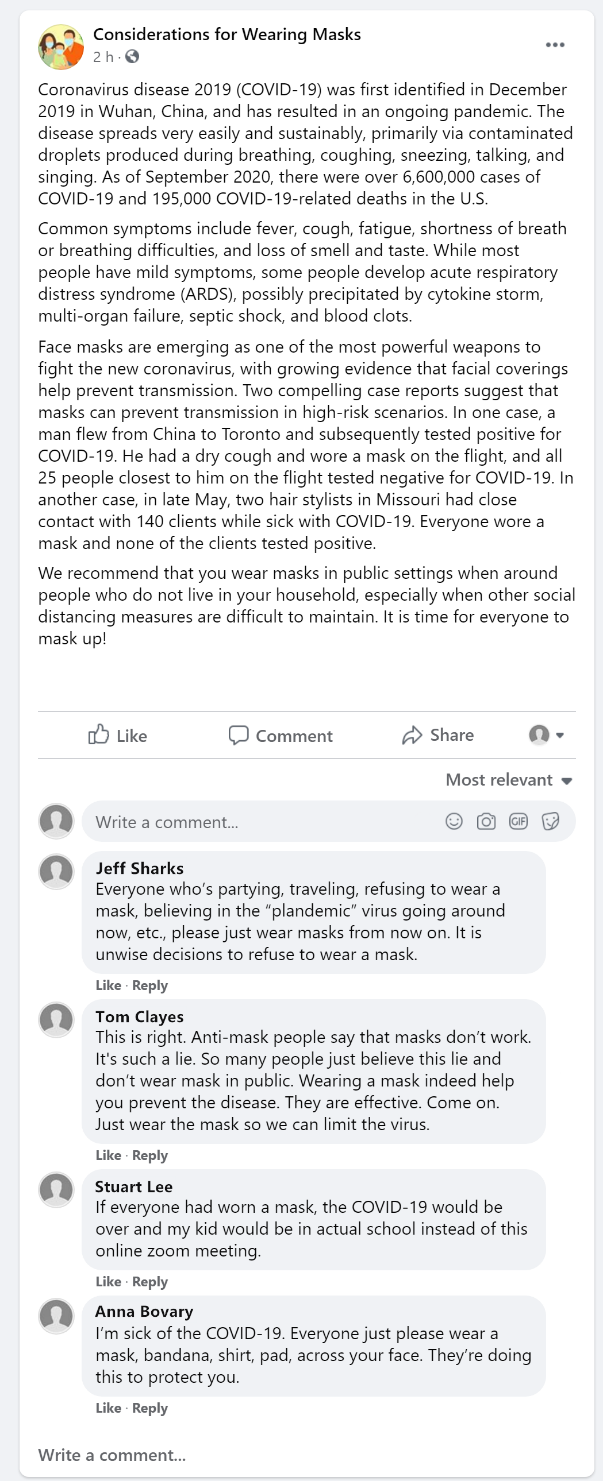

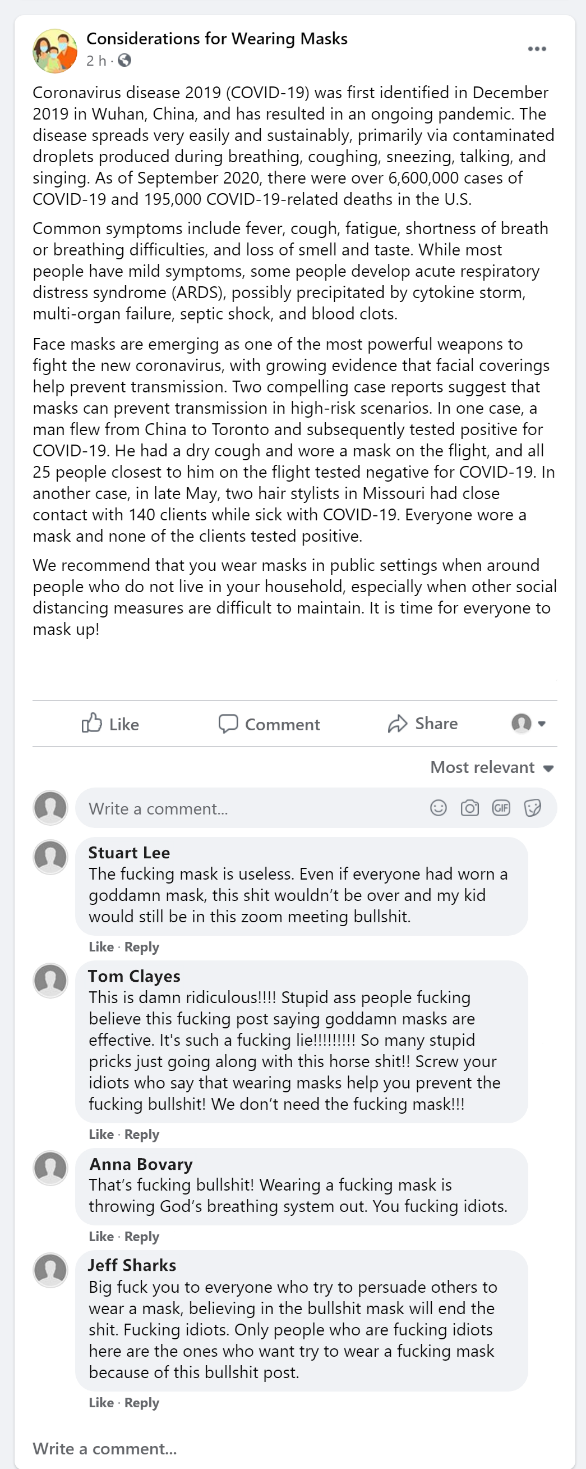

Supplement: Multimedia Appendix 1 [file jmir_v26i1e57967_app1.docx]
