# Supplementary material for: The Impact of Comment Slant and Comment Tone on Digital Health Communication Among Polarized Publics: A Web-Based Survey Experiment
Source: J Med Internet Res. 2024 Nov 15;26:e57967. doi: 10.2196/57967 (PMC11607566; doi:10.2196/57967)
Supplement: Multimedia Appendix 4 [file jmir_v26i1e57967_app4.docx]

|  | Group 1  (n = 130) | Group 2  (n= 129) | Group  (n = 131) | Group 4  (n = 132) | *P* |
| --- | --- | --- | --- | --- | --- |
| **Age (years), mean (SD)** | | | | |  |
|  | 40.84  (13.08) | 41.41  (11.45) | 42.45  (12.45) | 41.61  (12.45) | .77 |
| **Gender, n (%)** | | | | | |
| Female | 65(50.0) | 58(45.0) | 54(41.2) | 54(40.9) | .42 |
| Male | 65(50.0) | 71(55.0) | 77(58.8) | 78(59.1) |  |
| **Educational level, n (%)** | | | | |  |
| 8 through 11 years | 1(0.8) | 0(0) | 0(0) | 0(0) | .37 |
| 12 years or completed high school | 14(10.8) | 15(11.6) | 13(9.9) | 14(10.6) |  |
| Post high school training other than college | 10(7.7) | 6(4.7) | 1(0.8) | 6(4.5) |  |
| Some college | 25(19.2) | 39(30.2) | 30(22.9) | 35(26.5) |  |
| College graduate | 67(51.5) | 51(39.5) | 67(51.1) | 61(46.2) |  |
| Postgraduate | 13(10.0) | 18(14.0) | 20(15.3) | 16(12.1) |  |
| **Monthly family income, n (%)** | | | | |  |
| $0 -- $9,999 | 5(3.8) | 5(3.9) | 5(3.9) | 9(6.8) | .54 |
| $10,000 -- $14,999 | 4(3.1) | 5(3.9) | 6(3.9) | 7(5.3) |  |
| $15,000 -- $19,999 | 3(2.3) | 0(0) | 4(0) | 6(4.5) |  |
| $20,000 -- $34,999 | 27(20.8) | 21(16.3) | 25(16.3) | 23(17.4) |  |
| $35,000 -- $49,999 | 26(20.0) | 32(24.8) | 17(24.8) | 23(17.4) |  |
| $50,000 -- $74,999 | 29(22.3) | 27(20.9) | 39(20.9) | 28(21.2) |  |
| $75,000 -- $99,999 | 20(15.4) | 21(16.3) | 19(16.3) | 18(13.6) |  |
| $100,000 -- $199,999 | 14(10.8) | 14(10.9) | 14(10.9) | 15(11.4) |  |
| $200,000 or more | 2(1.5) | 4(3.1) | 2(3.1) | 3(2.3) |  |
| **Race, n (%)** | | | | | |
| White | 104(80.0) | 102(79.1) | 106(80.9) | 104(78.8) | .97 |
| Non-white | 26(20.0) | 27(20.9) | 25(19.1) | 28(21.2) |  |
| **Political identification, n (%)** | | | | |  |
| Democrats | 45(34.6) | 56(43.4) | 63(48.1) | 53(40.2) | .21 |
| Republicans | 85(65.4) | 73(56.6) | 68(51.9) | 79(59.8) |  |
| Neither republicans nor democrats | 23(17.7) | 19(14.7) | 24(18.3) | 29(22.0) |  |

*Note.* Group 1: civil anti-mask-wearing comments; Group 2: civil pro-mask-wearing comments; Group 3: uncivil anti-mask-wearing comments; Group 4: uncivil pro-mask-wearing comments. P value is the test in difference among groups.
