# Supplementary material for: The Impact of Comment Slant and Comment Tone on Digital Health Communication Among Polarized Publics: A Web-Based Survey Experiment
Source: J Med Internet Res. 2024 Nov 15;26:e57967. doi: 10.2196/57967 (PMC11607566; doi:10.2196/57967)
Supplement: Multimedia Appendix 5 [file jmir_v26i1e57967_app5.docx]

|  | **Presumed influence** | | **Behavioral intention to wear masks** | |
| --- | --- | --- | --- | --- |
|  | *B*(se) | *P*(95%CI) | *B*(se) | *P*(95%CI) |
| Age | -.00(.01) | .81(-0.01, 0.01) | .01(.00) | .19(-0.003, 0.013) |
| Gender | .04(.16) | .76(-0.23, 0.31) | .22(.10) | .02(0.03, 0.40) |
| Education | .13(.06) | .04(0.01, 0.25) | -.09(.04) | .03(-0.18, -0.01) |
| Income | -.11(.04) | .003(-0.18, -0.04) | .02(.03) | .37(-0.03, 0.08) |
| Race | -.10(.17) | .56(-0.43, 0.24) | .16(.12) | .19(-0.08, 0.39) |
| Republican | .15(.19) | .44(-0.23, 0.53) | -.01(.14) | .92(-0.28, 0.25) |
| Democrat | .52(.20) | .008(0.14, 0.90) | .04(.14) | .77(-0.23, 0.31) |
| Mask wearing frequency | .21(.07) | .001(0.09, 0.34) | 1.04(.05) | < .001(0.95, 1.13) |
| Social media use frequency | .13(.07) | .08(-0.02, 0.27) | .02(.05) | .73(-0.08, 0.12) |
| Attitude extremity | -0.05(0.09) | 0.59(-0.22, 0.13) | -0.34(0.06) | < .001(-0.46, -0.22) |
| Prior attitude (A) | .20(.29) | .48(-0.36, 0.76) | .96(.20) | < .001(0.57, 1.36) |
| Comment slant (S) | 1.49(.26) | < .001(0.98, 2.00) | -.10(.19) | .60(-0.47, 0.27) |
| Comment tone (T) | .64(.26) | .01(0.13, 1.15) | -.38(.18) | .04(-0.74, -0.03) |
| A * S | .27(.37) | .46(-0.46, 1.00) | -.12(.26) | .64(-0.63, 0.39) |
| A * T | -.27(.37) | .48(-1.00, 0.47) | .31(.26) | .24(-0.21, 0.83) |
| S * T | .08(.36) | .83(-0.64, 0.80) | .72(.26) | .005(0.21, 1.22) |
| A* S * T | .56(.53) | .29(-0.48, 1.60) | -.78(.37) | .04(-1.50, -0.05) |
| Presumed influence | -- | -- | .06(.03) | .04(0.003, 0.126) |
| Model summary | *F* (17, 504) = 17.86, *P*<.001 | | *F* (18, 503) = 68.53, *P*<.001 | |
