# Supplementary material for: The Impact of Comment Slant and Comment Tone on Digital Health Communication Among Polarized Publics: A Web-Based Survey Experiment
Source: J Med Internet Res. 2024 Nov 15;26:e57967. doi: 10.2196/57967 (PMC11607566; doi:10.2196/57967)
Supplement: Multimedia Appendix 6 [file jmir_v26i1e57967_app6.docx]

|  | Indirect effects on behavioral intention | | | Direct effects on behavioral intention | | |
| --- | --- | --- | --- | --- | --- | --- |
|  | Effect | SE | 95% CI | Effect | SE | 95% CI |
| Comment Slant | | | | | | |
| Uncivil * Anti-maskers | .10 | .05 | [.003, .202] | -.10 | .19 | [-.47, .27] |
| Uncivil * Pro-maskers | .11 | .06 | [.004, .239] | -.22 | .19 | [-.60, .16] |
| Civil * Anti-maskers | .10 | .05 | [.003, .211] | .62 | .19 | [.25, 0.98] |
| Civil * Pro-maskers | .15 | .08 | [.004, .310] | -.28 | .20 | [-.68, .13] |
| Comment Tone | | | | | | |
| Anti-mask-wearing * Anti-maskers | .04 | .03 | [-.00, .11] | -.38 | .18 | [-.74, -.03] |
| Anti-mask-wearing * Pro-maskers | .02 | .03 | [-.02, .09] | -.08 | .19 | [-.45, .30] |
| Pro-mask-wearing * Anti-maskers | .05 | .03 | [.002, .115] | .33 | .18 | [-.03, .69] |
| Pro-mask-wearing * Pro-maskers | .07 | .03 | [.004, .140] | -.13 | .19 | [-.51, .24] |
| Prior attitudes | | | | | | |
| Anti-mask-wearing * Uncivil | .01 | .02 | [-.03, .07] | .96 | .20 | [.57, 1.36] |
| Anti-mask-wearing * Civil | -.00 | .02 | [-.05, .04] | 1.27 | .20 | [.88, 1.66] |
| Pro-mask-wearing * Uncivil | .03 | .03 | [-.01, .09] | .84 | .19 | [.46, 1.22] |
| Pro-mask-wearing * Civil | .05 | .03 | [.002, .111] | .37 | .20 | [-.01, .76] |
