# Supplementary material for: The Impact of Comment Slant and Comment Tone on Digital Health Communication Among Polarized Publics: A Web-Based Survey Experiment
Source: J Med Internet Res. 2024 Nov 15;26:e57967. doi: 10.2196/57967 (PMC11607566; doi:10.2196/57967)
Supplement: Multimedia Appendix 7 [file jmir_v26i1e57967_app7.docx]

|  | Presumed influence | | | | | |
| --- | --- | --- | --- | --- | --- | --- |
|  | Model 1 | | Model 2 | | Model 3 | |
|  | *B*(se) | *P* | *B*(se) | *P* | *B*(se) | *P* |
| Age | -.00(.01) | .81 | .01(.00) | .19 | -.00(.01) | .81 |
| Gender | .04(.16) | .76 | .22(.10) | .02 | .03(.14) | .81 |
| Education | .13(.06) | .04 | -.09(.04) | .03 | .13(.06) | .04 |
| Income | -.11(.04) | .003 | .02(.03) | .37 | -.11(.04) | .003 |
| Race | -.10(.17) | .56 | .16(.12) | .19 | -.09(.17) | .59 |
| Republican | .15(.19) | .44 | -.01(.14) | .92 | .15(.19) | .43 |
| Democrat | .52(.20) | .008 | .04(.14) | .77 | .52(.20) | .008 |
| Mask wearing frequency | .21(.07) | .001 | 1.04(.05) | < .001 | .22(.06) | < .001 |
| Social media use frequency | .13(.07) | .08 | .02(.05) | .73 | .13(.07) | .08 |
| Prior attitude (A) | -- | -- | .96(.20) | < .001 | .19(.28) | .51 |
| Comment slant (S) | -- | -- | -.10(.19) | .60 | 1.50(.26) | < .001 |
| Comment tone (T) | -- | -- | -.38(.18) | .04 | .63(.26) | .02 |
| A * S | -- | -- | -- | -- | .27(.37) | .47 |
| A * T | -- | -- | -- | -- | -.26(.37) | .48 |
| S * T | -- | -- | -- | -- | .09(.36) | .80 |
| A* S * T | -- | -- | -- | -- | .55(.53) | .30 |
| Model summary | *F* (9, 512) = 5.67, *P* <.001 | | *F* (12, 509) = 24.57, *P* <.001 | | *F* (16, 505) = 18.99, *P* <.001 | |
