# Supplementary material for: The Impact of Comment Slant and Comment Tone on Digital Health Communication Among Polarized Publics: A Web-Based Survey Experiment
Source: J Med Internet Res. 2024 Nov 15;26:e57967. doi: 10.2196/57967 (PMC11607566; doi:10.2196/57967)
Supplement: Multimedia Appendix 8 [file jmir_v26i1e57967_app8.docx]

|  | Behavioral intention to wear masks | | | | | | | |
| --- | --- | --- | --- | --- | --- | --- | --- | --- |
|  | Model 1 | | Model 2 | | Model 3 | | Model 4 | |
|  | *B*(se) | *P* | *B*(se) | *P* | *B*(se) | *P* | *B*(se) | *P* |
| Age | .01(.00) | .11 | .01(.00) | .16 | .01(.00) | .20 | .01(.00) | .19 |
| Gender | .12(.10) | .26 | .16(.10) | .11 | .17(.10) | .09 | .16(.10) | .09 |
| Education | -.05(.05) | .32 | -.08(.05) | .09 | -.09(.05) | .06 | -.09(.05) | .04 |
| Income | .01(.03) | .79 | .02(.03) | 50 | .02(.03) | .57 | .02(.03) | .40 |
| Race | .23(.13) | .07 | .18(.12) | .15 | .20(.12) | .11 | .20(.12) | .10 |
| Republican | -.13(.15) | .38 | -.01(.14) | .97 | .03(.14) | .85 | .02(.14) | .91 |
| Democrat | .19(.15) | .19 | .06(.14) | .66 | .07(.14) | .63 | .03(.14) | .82 |
| Mask wearing frequency | 1.23(.04) | <.001 | 1.10(.05) | < .001 | 1.09(.05) | < .001 | 1.08(.05) | < .001 |
| Social media use frequency | .01(.06) | .90 | .02(.05) | .76 | .02(.05) | .74 | .01(.05) | .87 |
| Prior attitude (A) | -- | -- | .76(.12) | < .001 | .87(.21) | < .001 | .86(.21) | < .001 |
| Comment slant (S) | -- | -- | .18(.10) | .06 | .04(.19) | .84 | -.06(.19) | .74 |
| Comment tone (T) | -- | -- | -.05(.10) | .64 | -.40(.19) | .03 | -.44(.19) | .02 |
| A * S | -- | -- | -- | -- | -.13(.27) | .63 | -.15(.27) | .58 |
| A * T | -- | -- | -- | -- | .32(.27) | .24 | .34(.27) | .21 |
| S * T | -- | -- | -- | -- | .80(.26) | .003 | .79(.26) | .003 |
| A* S * T | -- | -- | -- | -- | -.80(.38) | .04 | -.84(.38) | .03 |
| Presumed influence | -- | -- | -- | -- | -- | -- | .07(.03) | .03 |
| Model summary | *F* (9, 512) = 5.67, *P*<.001 | | *F* (12, 509) = 24.57, *P*<.001 | | *F* (16, 505) = 18.99, *P*<.001 | | *F* (17, 504) = 67.01, *P*<.001 | |
